# Acute Liver Failure Prognostic Criteria: It's Time to Revisit

**DOI:** 10.7759/cureus.33810

**Published:** 2023-01-16

**Authors:** Amit Goel, David Lalruatsanga, D Himanshu, Vipin Bharti, Deepak Sharma

**Affiliations:** 1 Gastroenterology, Sanjay Gandhi Postgraduate Institute of Medical Sciences (SGPGI), Lucknow, IND; 2 Internal Medicine, Zoram Medical College, Mizoram, IND; 3 Internal Medicine, King George's Medical University, Lucknow, IND

**Keywords:** liver transplant, prognostic marker, clif-sofa score, meld score, acute liver failure

## Abstract

Introduction: Acute liver failure (ALF) is a devastating disease, and patients are at a higher risk of death without liver transplantation. Indicators are needed to identify the risk of death in ALF, which will help in the timely referral of patients to specialized centers. Clichy criteriaand King’s College Hospital (KCH) criteria are the most widely used prognostic criteria. Real-life application of Clichy criteria is limited due to the non-availability of factor V level measurement. KCH criteria have good specificity but low sensitivity to predict outcomes. Therefore, we attempted to use the model for end-stage liver disease (MELD) score and chronic liver failure-sequential organ failure assessment (CLIF-SOFA) score in ALF patients as prognostic indicators and need for liver transplantation.

Methods: Forty-one patients with ALF were enrolled in the study. On the day of admission, MELD and CLIF-SOFA scores were calculated for each patient. Area under receiver operating characteristics (AUROC) curve, sensitivity, specificity, negative predictive value (NPV), positive predictive value (PPV), and diagnostic accuracy (DA) of MELD and CLIF-SOFA score were calculated to predict the outcome of the patients.

Results: Out of 41 patients, nine patients left against medical advice. The sensitivity, specificity, PPV, NPV, and DA for the MELD score of enrolled patients in the study were 81.5%, 62.5%, 59.5%, 83.3%, 70.1%, and for the CLIF-SOFA score of enrolled patients in the study were 88.9%, 90.0%, 85.7%, 92.3%, 89.6% respectively. Patients who did not survive had higher INR, MELD, CLIF-SOFA scores, and hepatic encephalopathy (HE) grades. Five patients who had a combination of MELD ≥30 and CLIF-SOFA ≥10, expired.

Conclusion: In our study, we used MELD score and CLIF-SOFA as prognostic markers, and we concluded that CLIF-SOFA is a better predictor of mortality than MELD score in terms of sensitivity, specificity, NPV, PPV, and diagnostic accuracy. AUROC for CLIF-SOFA score is higher when compared to the MELD score.

## Introduction

Severe liver injury in a short period of time may result in acute liver failure (ALF). Liver injury is marked by the elevation of liver enzymes in serum. Progression of liver injury to ALF is marked with hepatic encephalopathy and coagulopathy. In the lack of liver transplantation, ALF patients are at high risk of death [1]. Identification of indicators for risk of death among those with ALF is needed. Such indicators will help in timely referral to specialized centers, reducing ALF-related deaths and appropriate prioritization of resource allocation.

Clichy criteria and King’s College Hospital (KCH) criteria are the most widely used prognostic criteria [2,3]. Clichy criteria recommend liver transplantation (LT) in patients with grade 3 or 4 encephalopathies and coagulation factor V level <20% (if age <30 years) or <30% (if age >30 years) [2,4]. Real-life application of Clichy criteria is limited due to the non-availability of factor V level measurement and their validation is restricted to hepatitis B reactive patients only. The King’s College Hospital criteria (KCH) remained the most widely used criteria for the prognosis of ALF patients. In 1989, the description of KCH criteria puts a step forward in identifying candidates for liver transplantation. KCH criteria stressed the importance of etiology and mode of presentation in the outcome of ALF. Poor sensitivity of this criteria indicates that a significant number of cases not fulfilling criteria would progress and die without earlier identification and consideration for possible liver transplantation. Few recent studies attempted to validate the KCH criteria and found it to have good specificity but low sensitivity to predict outcomes [5-8].

Search is continued for newer models with better diagnostic performance. Therefore, we attempted to use the model for end-stage liver disease (MELD) score and chronic liver failure-sequential organ failure assessment (CLIF-SOFA) score in ALF patients as prognostic indicators and need for liver transplantation.

## Materials and methods

A prospective observational study was conducted in the department of Internal Medicine, King George’s Medical University (KGMU), Lucknow, India. Ethical clearance for this study was approved by the Institutional Ethics Committee, KGMU, (reference code: 99th ECM II B-thesis/P3). Participants admitted with ALF between August 2018 and July 2019 were screened for eligibility criteria. ALF was diagnosed with Asia Pacific Association for the Study of Liver (APASL) criteria, i.e., total serum bilirubin (TSB) >5 mg%, coagulopathy (INR >1.5), hepatic encephalopathy within 4 weeks of Illness in a person without the pre-existing liver disease [9]. The study included all patients above the age of 13 years with features of ALF as per APASL guidelines. Patients not giving consent or withdrawing consent, primary non-function of liver graft or graft dysfunction, known malignancies, ischemic hepatitis, patients with clinical suspicion of brain death (confirmed by a neurologist), and patients with intrinsic renal disease were excluded from the study.

For each patient MELD and CLIF-SOFA score (Table 1) was calculated on the day of admission. MELD uses the patient's values for serum bilirubin, serum creatinine, and the international normalized ratio for prothrombin time (INR) to predict survival. It is calculated according to the following formula: MELD = 3.78×ln(serum bilirubin {mg/dL}) + 11.2×ln(INR) + 9.57×ln(serum creatinine {mg/dL}) + 6.43. All those included in the study were managed with the standard of care recommended for the management of ALF [10]. Continuous data were summarized as Mean ± SE (standard error of the mean) whereas discrete (categorical) data was summarized in number (n) and percentage (%). Student’s t-test compared continuous two independent groups. Continuous data from two dependent groups were compared by paired t-test. Continuous data (more than two independent groups) were compared by one-factor analysis of variance (ANOVA) and the significance of the mean difference between (inter) the groups was done by Tukey’s HSD (honestly significant difference) post hoc test after ascertaining normality by Shapiro-Wilk’s test and homogeneity of variance between groups by Levene’s test. Categorical groups were compared by the Chi-square (χ2) test. A two-tailed (α=2) p<0.05 was considered statistically significant. Analyses were performed on SPSS software, Windows version 17.0 (SPSS Inc., Chicago). We calculated the area under receiver operating characteristics (AUROC) curves, sensitivity, specificity, negative predictive value (NPV), positive predictive value (PPV), and diagnostic accuracy (DA) of MELD and CLIF-SOFA scores to predict the final outcome.

**Table 1:** Chronic liver failure-sequential organ failure assessment score HE: hepatic encephalopathy, INR: international normalized ratio, MAP: mean arterial pressure, PaO_2_: partial pressure of arterial oxygen, FiO_2_: fraction of inspired oxygen, SpO_2_: Saturation of Peripheral Oxygen, DA: dopamine, DOB: dobutamine, E: epinephrine, NE: norepinephrine, PLT: platelet count

Organ failure	Score=0	Score=1	Score=2	Score=3	Score=4
Liver: Total bilirubin (mg/dL)	< 1.2	≥ 1.2 to < 2.0	≥ 2.0 to < 6.0	≥ 6.0 to < 12	≥ 12.0
Kidney: Serum creatinine (mg/dL)	< 1.2	≥ 1.2 to < 2.0	≥ 2.0 to < 3.5	≥ 6.0 to < 12	≥ 5.0
	Or use of renal replacement therapy				
Cerebral (HE grade)	Absent	I	II	III	IV
Coagulation (INR)	< 1.1	≥ 1.1 to < 1.25	≥ 1.25 to < 1.5	≥ 1.5 to < 2.5	≥ 2.5 or PLT ≤ 20 × 10^9^/L
Circulation (MAP, mmHg)	≥ 70	< 70	DA ≤ 5 or DOB or Terlipressin	DA > 5 or E ≤ 0.1 or NE ≤ 0.1	DA > 15 or E 0.1 or NE > 0.1
Lung PaO_2_/FiO_2_	> 400	> 300 to ≤ 400	> 200 to ≤ 300	> 100 to ≤ 200	≤ 100
Or SpO_2_/FiO_2_	> 512	> 357 to ≤ 512	> 214 to ≤ 357	> 89 to ≤ 214	≤ 89

## Results

We included 41 patients (males 24; 58.5%). The most common etiology identified for ALF was hepatitis B (19; 46.3%), followed by hepatitis E (10; 24.4%), hepatitis A (3, 7.3%), and autoimmune hepatitis (2, 4.9%). The outcomes of the nine participants (22%) were not known as they had left the hospital against medical advice. Data from 32 participants were included for analysis. Of the remaining 32 participants, twelve (29.3%) died. The non-survivors had higher INR, MELD score, CLIF-SOFA score, and grades of HE as compared to survivors (Table 2).

**Table 2:** Baseline characteristics of survivors and non-survivors on admission in patients with acute liver failure MELD: model for end-stage liver disease, CLIF-SOFA: chronic liver failure-sequential organ failure assessment, HE: hepatic encephalopathy

Variable	Survivor (n=20)	Non-Survivor (n=12)	p-value
Age	28.62±9.30	32.92±12.33	0.265
Gender (Female/Male)	8/12	5/7	0.947
HE Grades 3 or 4	8 (40%)	12 (60%)	<0.001
Total Bilirubin	15.8±7.23	21.4±9.00	0.060
International normalized ratio (INR)	2.39±0.92	3.43±1.21	0.009
S. Creatinine	1.11±0.77	1.41±0.71	0.291
Platelet count	1.88±0.91	1.59±0.77	0.372
MELD	27.00±5.07	34.17±6.16	0.001
CLIF-SOFA	6.95±1.43	9.83±1.11	<0.001

Of the 11 participants who had a MELD score ≥30 at admission, eight (72.2%) died. Similarly, among eight patients with CLIF-SOFA score ≥10 at admission, seven expired (87.5%). All five patients, who had a combination of MELD ≥30 and CLIF-SOFA ≥10, expired.

**Figure 1:**
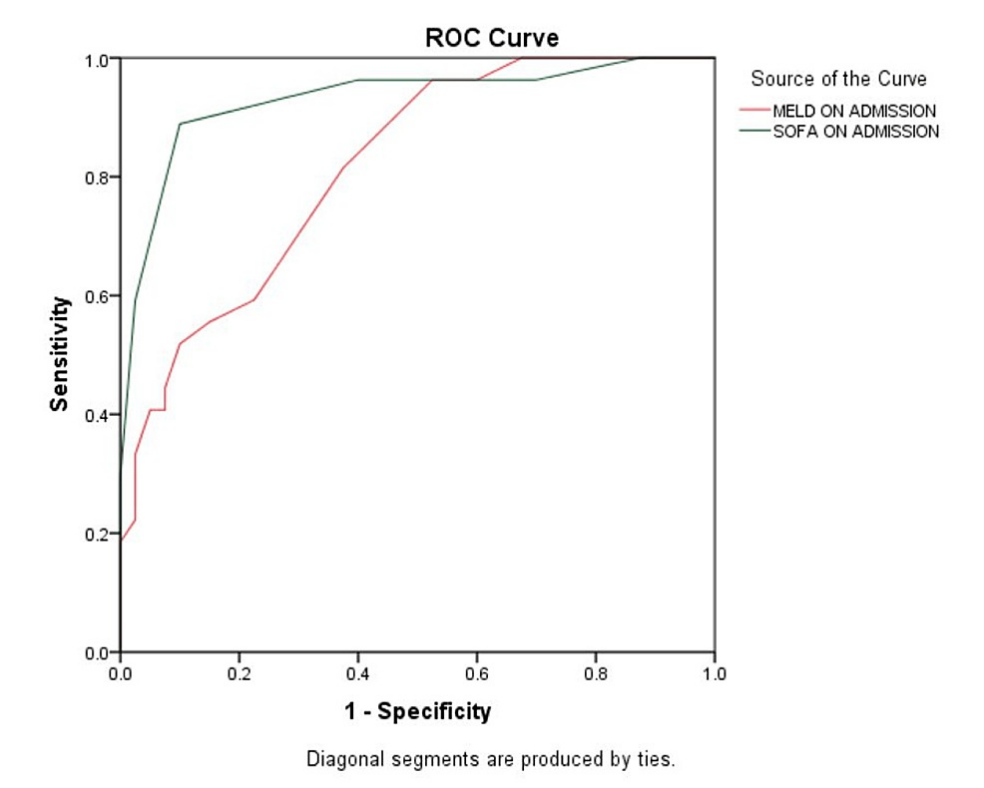
Receiver operating curve (ROC) for the analysis of MELD and CLIF-SOFA score for the prediction of mortality MELD: model for end-stage liver disease, CLIF-SOFA: chronic liver failure-sequential organ failure assessment

The receiver operating characteristics (ROC) analysis showed the CLIF-SOFA score as a better predictor in comparison to the MELD score (Figure 1). The AUROC SOFA > AUROC MELD. The cut-off for predicting mortality by MELD was estimated to be ≥28.5 with sensitivity, specificity, positive predictive value (PPV), negative predictive value (NPV), and diagnostic accuracy (DA) as 81.5%, 62.5%, 59.5%, 83.3%, and 70.1%. The cut-off for predicting mortality by CLIF-SOFA was estimated to be ≥8.5 with sensitivity, specificity, PPV, NPV, and DA as 88.9%, 90.0%, 85.7%, 92.3%, and 89.6% (Table 3).

**Table 3:** Comparison of MELD and CLIF-SOFA on the basis of sensitivity, specificity, positive predictive value (PPV), negative predictive value (NPV), and diagnostic accuracy in acute liver failure

Parameter	MELD	CLIF-SOFA
Area under the curve	0.816	0.930
Optimum cut-off for mortality	≥28.5	≥8.5
Sensitivity	81.5	88.9
Specificity	62.5	90.0
Positive predictive value	59.5	85.7
Negative predictive value	83.3	92.3
Diagnostic accuracy	70.1	89.6

## Discussion

The model for end-stage liver disease (MELD) is a composite score derived from three laboratory parameters, namely total serum bilirubin, serum creatinine, and international normalized ratio (INR). MELD is widely used to predict short-term mortality among patients with cirrhosis who are on the waiting list for liver transplantation [9]. Data from the United Network for Organ Sharing (UNOS) status 1 listed patients, suggests that a high MELD score is a predictor of death, and therefore suggests an urgent need for liver transplantation, in non-acetaminophen-induced ALF [11]. MELD alongside its components (serum bilirubin, serum creatinine, and INR) have also been used for the prognosis of ALF. A higher grade of HE was found to be a predictor of mortality in various studies [11,12].

SOFA score, which is commonly used in the intensive care setting, was recently modified by the EASL-CLIF Consortium to predict mortality in ACLF patients admitted to ICUs. This score includes failure of six organ systems (cerebral, hepatic, respiratory, coagulopathy, circulatory and renal), with the severity of their failure scored from 0 to 4, for a total score between 0 and 24 [13].

Patients with ALF have a high risk of death without LT and also need to be managed in the ICU setting. In a resource-poor country, such as India, LT is not an option for every patient who needs it and access to ICU care is also limited. Hence, we need prognostic indices to prioritize those who are at a higher risk for death. Such prioritization helps in early referral to higher centers and thoughtful allocation of scarce ICU resources. An ideal prognostic marker is one that can be used at the bedside, is reliable, reproducible, rapidly and accurately measures the change in clinical status, includes objective parameters, and identifies before the multi-organ dysfunction has set in.

Originally MELD score was derived to estimate the short-term survival of patients undergoing trans jugular intra-hepatic postoperative shunts [14] and is widely used to prioritize cirrhosis patients for liver transplantation [9]. The most common etiology for ALF in our population was viral hepatitis whereas acetaminophen overdose is the most common in western countries. Previous studies from India have also shown viral etiology as the most common etiology for ALF [15].

We enrolled 41 patients in this study. Nine patients left the hospital against medical advice. Data from 32 patients were included for analysis. The most common etiology identified as hepatitis B (19; 46.3%). The mortality rate in the study population was 37.5%. patients who did not survive had higher INR, HE grades, MELD scores, and CLIF-SOFA scores. 

Kamath et al. concluded that patients with MELD > 30 had the highest mortality [16]. Saluja et al. have demonstrated that patients with ALF and MELD > 30 are at the greatest risk for mortality in the first three or four days after listing. Similar to previous results from several investigators, we also found that MELD >30 was associated with a very high risk of death [17].

Hepatic encephalopathy was found to be a predictor of mortality in various studies. Xiong et al. [18] demonstrated that a hepatic encephalopathy of grade 3 or 4 was associated with poor outcomes, and Mainardi et al. [12] concluded that the mortality rate is 94% in patients with a higher grade of hepatic encephalopathy (grade 3 or 4). In this study mortality rate is 60.0% in grade 3 or grade 4 hepatic encephalopathy in patients with ALF.

CLIF-SOFA score correlates best with the final outcome of the patients having ALF as demonstrated by various literature all over the world. Cholongitas et al. [19] showed that the SOFA score provided the best discriminative ability in acetaminophen-induced liver failure. Saluja et al. [17] concluded that among various prognostic scores for ALF, SOFA 48 hours have performed the best. More such studies are required in patients with ALF to validate such outcomes.

Limitations: Better outcomes can be expected with a larger sample size. Acetaminophen-related ALF is not common in the Indian scenario, so it contributes less as an etiological agent of ALF as compared to than western world. Since our institution is a tertiary care center, the patients are referred to us in low general condition and advanced stages of hepatic encephalopathy which contribute to poor outcomes in such patients.

## Conclusions

In our study, we used the MELD score and CLIF-SOFA as prognostic markers and concluded that CLIF-SOFA is a better predictor of mortality than the MELD score in terms of sensitivity, specificity, NPV, PPV, and diagnostic accuracy. AUROC for CLIF-SOFA score is higher when compared to the MELD score. This could be explained by the fact that the CLIF-SOFA score includes six functional organ failures (cerebral, hepatic, respiratory, coagulopathy, circulatory and renal). Further studies are needed to validate that the CLIF-SOFA score is as good as other prognostic scores used to predict bedside mortality and the need for liver transplantation in ALF.
